# Recruitment, Retainment, and Biomarkers of Response; A Pilot Trial of Lithium in Humans With Mild Cognitive Impairment

**DOI:** 10.3389/fnmol.2019.00163

**Published:** 2019-06-28

**Authors:** Ashleigh Duthie, Lidy van Aalten, Cara MacDonald, Alison McNeilly, Jennifer Gallagher, John Geddes, Simon Lovestone, Calum Sutherland

**Affiliations:** ^1^Ninewells Hospital and Medical School, NHS Tayside, Dundee, United Kingdom; ^2^Division of Cellular Medicine, University of Dundee, Dundee, United Kingdom; ^3^Department of Psychiatry, Warneford Hospital, University of Oxford, Oxford, United Kingdom

**Keywords:** lithium, GSK3, clinical trial, biomarker, safety

## Abstract

Lithium has been used for decades to treat Bipolar Disorder. Some of its therapeutic benefits may be through inhibition of Glycogen Synthase Kinase (GSK)-3. Enhanced GSK3 activity associates with development of Alzheimer’s disease (AD), therefore lithium is a currently used therapeutic with potential to be repurposed for prevention of Dementia. An important step toward a clinical trial for AD prevention using lithium is to establish the dose of lithium that blocks GSK3 in Mild Cognitive Impairment (MCI), a high-risk condition for progression to AD. We investigated volunteer recruitment, retention, and tolerance in this population, and assessed biomarkers of GSK3 in MCI compared to control and after lithium treatment. Recruitment was close to target, with higher than anticipated interest. Drop out was not related to lithium blood concentration. Indeed, 33% of the withdrawals were in the first week of very low dose lithium. Most made it through to the highest dose of lithium with no adverse events. We analyzed 18 potential biomarkers of GSK3 biology in rat PBMCs, but only four of these gave a robust reproducible baseline signal. The only biomarker that was modified by acute lithium injection in the rat was the inhibitory phosphorylation of Ser9 of GSK3beta (enhanced in PBMCs) and this associated with reduced activity of GSK3beta. In contrast to the rat PBMC preparations the protein quality of the human PBMC preparations was extremely variable. There was no difference between GSK3 biomarkers in MCI and control PBMC preparations and no significant effect of chronic lithium on the robust GSK3 biomarkers, indicating that the dose reached may not be sufficient to modify these markers. In summary, the high interest from the MCI population, and the lack of any adverse events, suggest that it would be relatively straightforward and safe to recruit to a larger clinical trial within this dosing regimen. However, it is clear that we will need an improved PBMC isolation process along with more robust, sensitive, and validated biomarkers of GSK3 function, in order to use GSK3 pathway regulation in human PBMC preparations as a biomarker of GSK3 inhibitor efficacy, within a clinical trial setting.

## Introduction

The pharmacological treatment of Dementia is limited to the treatment of symptoms, primarily due to the ever increasing number of failed clinical trials (Hardy, 2009; Wang et al., 2016; Amanatkar et al., 2017), while the paucity of non-amyloid targeting drugs in development is highly concerning (Cummings et al., 2017). In contrast, there is increasing awareness of the importance of identifying high risk groups and developing Dementia prevention strategies.

Mild Cognitive impairment (MCI) can be thought of as a prodromal phase of Dementia with a significant proportion of MCI patients converting to Dementia. Interventions in MCI may provide the best opportunities to delay or prevent the development of irreversible pathology and debilitating symptoms (Petersen et al., 2001). In order to generate novel therapeutics as rapidly as possible one approach is to investigate the “repositioning” of therapeutics currently used for other neurological conditions, with acceptable safety and side effect profiles, that have mechanistic potential to target the molecular pathways in brain associated with early AD development (e.g., amyloid, tangle, inflammation, etc.) (Corbett et al., 2012).

Glycogen Synthase Kinase-3 (GSK3) influences many important cellular processes (Jope and Johnson, 2004; Kockeritz et al., 2006; Sutherland, 2011), and its dysregulation has been linked to the pathophysiology of numerous age-related diseases including Alzheimer’s disease (AD) and inflammation (Cohen and Goedert, 2004; O’Brien and Klein, 2007; Hooper et al., 2008; Sutherland, 2011; Gao et al., 2012; Beurel et al., 2013; Russo et al., 2013). Importantly, partial deletion (pharmacological or genetic) of GSK3 reduces the severity of disease in many preclinical models, including models of familial AD (Jope et al., 2007; Hooper et al., 2008; Sutherland, 2011; Avrahami et al., 2013; Ly et al., 2013). Furthermore, transgenic overexpression of GSK3, specifically in mouse brain, promotes AD-like symptoms and generates pathology (Lucas et al., 2001). Importantly, GSK3 activation is directly linked to the development of cognitive impairment independent of AD pathology (King et al., 2013). This implies that GSK3 inhibition could have clinical benefits from early stage development right through to late stage disease. However, the wide spectrum of important biological actions influenced by GSK3 activity has questioned the clinical safety of a global GSK3 inhibition approach, especially as genetic ablation of GSK3 has serious health implications including disruption of brain development and repair (Jiang et al., 2005; Kim et al., 2009). However, GSK3 in human dementia is related to abnormally high GSK3 activity in adults, and no GSK3 inhibitor toxicology studies have been reported in an adult preclinical model with excessive GSK3 activity.

The safety concerns around GSK3 inhibition are doubly surprising since a GSK3 inhibitor is in everyday clinical use without the side-effects observed in some animal models of GSK3 ablation. Lithium is a well characterized (if non-selective) inhibitor of GSK3 (Klein and Melton, 1996; O’Brien and Klein, 2007, 2009; Can et al., 2014) with a well-known and acceptable side effect profile. Indeed, it has been used effectively for decades in the treatment of mood disorder, and although the precise mechanisms of action remain the subject of some debate there is evidence for at least some of its neuroprotective actions being mediated by inhibition of GSK3 (O’Brien and Klein, 2007, 2009; Malhi et al., 2013; Can et al., 2014). Importantly, patients prescribed long term lithium for Bipolar disorder have lower incidence of all forms of dementia compared to short term use or alternative therapies (Sutherland and Duthie, 2015). This is tantalizing evidence that lithium, or other GSK3 inhibitors, have the potential to prevent conversion to dementia.

Lithium is generally well tolerated as a long-term therapy for Bipolar disease in humans. However, toxicity can lead to ataxia and confusion, and in severe cases it can be life threatening. For this reason, lithium serum levels are routinely monitored in clinical practice and kept within a narrow therapeutic range of 0.4–0.75 mM/l (McKnight et al., 2012). Clinical studies giving lithium to elderly people with AD (at doses relevant to treatment of Bipolar disease) had relatively few side effects (Macdonald et al., 2008; Hampel et al., 2009). A study with lithium in people with MCI reported excellent volunteer retention and tolerability up to 1 year of lithium administration (Forlenza et al., 2011a). This treatment indicated a trend toward cognitive benefit and 91% tolerability but was too short to include effects on conversion to Dementia (Forlenza et al., 2011a). It seems reasonable therefore to suggest that lithium is a clinically viable GSK3 inhibitor that is available for immediate use in trials to evaluate GSK3 inhibition as an anti-dementia therapeutic agent.

The therapeutic range of lithium contains the IC50 (50% inhibition constant) for direct GSK3 inhibition *in vitro* [approx. 0.5 mM (Davies et al., 2000)], suggesting that pharmacologically relevant GSK3 inhibition would be achieved by the doses used to treat Bipolar disease. However, lithium regulates GSK3 activity by several distinct mechanisms (Sutherland and Duthie, 2015), which may mean that inhibition of GSK3 in patients will occur with even lower levels of lithium than predicted from *in vitro* obtained IC50 values. Therefore, experimentally evaluating the lithium concentration required to inhibit GSK3 in relevant human populations becomes a valuable exercise prior to balancing safety concerns with therapeutic benefits. However, measuring changes in GSK3 activity in humans is challenging, and almost impossible in intact brain.

The studies where lithium administration to rodent models of dementia has beneficial actions on brain imaging, behavior, or AD pathology [for example (O’Brien et al., 2004; Noble et al., 2005; Zhang et al., 2011; Sudduth et al., 2012; Yu et al., 2012; Chen et al., 2013; de Cristobal et al., 2014)] measure the N-terminal inhibitory phosphorylation status of GSK3 in a tissue lysate to assess GSK3 activity status. However, this modification does not inhibit GSK3 against all of its downstream targets (Frame et al., 2001), and thus is not sufficient to provide a comprehensive assessment of GSK3 activity. In reality, GSK3 inhibition may occur at lithium concentrations lower than those required to regulate this phosphorylation or to competitively inhibit GSK3 activity, and specific targets may be regulated by different mechanisms of GSK3 regulation (Robertson et al., 2018). There is evidence that therapeutic doses of lithium are sufficient to increase GSK3 N-terminal phosphorylation in human blood cells in Bipolar disorder (Li et al., 2007) or MCI (Forlenza et al., 2011b).

Alternative “potential biomarkers” of GSK3 activity status are detailed in Table 1. There are several phosphorylation sites on GSK3 that influence GSK3 activity, including Tyr 279/216 and Thr390 (GSK3β only) (Table 1), and total GSK3 isoform mRNA or protein can also vary in some disease states. One can also monitor the signaling pathways that regulate phosphorylation of these sites, such as the PI3K-Akt pathway (Cross et al., 1995). Activation of these pathways may be extrapolated to an assumption of inhibition of GSK3 (Table 1). Possibly more informative is quantitative assessment of phosphorylation of direct downstream targets of GSK3 (Table 1; Sutherland, 2011). However, this depends on antibody availability and protein detection in the tissues of interest.

**Table 1:** Potential molecular biomarkers of GSK3 activity, classified by the relationship to GSK3 function.

**(1) GSK3 regulatory modifications**	**Effect of modification**
Ser9 (GSK3b)/Ser21 (GSK3a) phosphorylation	Inhibits GSK3 activity toward primed substrates
Tyr216 (GSK3b)/Tyr279 (GSK3a) phosphorylation	Required for GSK3 kinase activity
Thr390 (GSK3b only)	Inhibits GSK3b activity
**(2) Downstream targets (effect of phosphorylation)**	**Residues phosphorylated by GSK3**
Glycogen Synthase (inhibited)	Ser641/645/649/653 (inhibits)
b-catenin (degraded)	Ser33/37 (degradation-so total b-catenin also a potential marker)
CRMP2 (cell location change)	Thr509/514/518
CRMP4 (cell location change)	Thr509/514/518
Tau (unknown but disease associated)	Ser231 Ser396 or PHF1(multiple)
**(3) Upstream regulatory pathways**	**Sites that monitor pathway**
PI-3K-PKB (main pathway for GSK3 inhibition through Ser9/21)	Thr308 and Ser473 PKB (active)
mTOR-S6K (has ability to inhibit GSK3 at Ser9/21)	Ribosomal S6 protein Ser240/244 (active)
RAS-MAPkinase (has ability to inhibit GSK3 at Ser9/21)	Thr180-Tyr182 p42/44 MAPK (active)
PKA (has ability to inhibit GSK3 at Ser9/21)	VASP (PKA target)
P38MAPK (inhibits GSK3b at Thr390)	ATF2 or Thr180-Tyr182 p38 MAPK (active)
**(4) Direct measure of GSK3**	
Immunoprecipitation and assay (GSK3a/b)	
Total GSK3a/b	

The current study aimed to assess and validate many of the potential biomarkers for GSK3 activity in Table 1, in rodent Peripheral Blood Mononuclear cells (PBMCs). We then took robust biomarkers from that study to generate pilot information on inhibition of GSK3 by lithium in people with MCI. Importantly we also aimed to extend the evidence that recruiting and retaining this population to a trial which involved taking lithium, a drug not normally prescribed to MCI, was achievable. We performed three studies, one where we gave rats an acute dose of lithium and measured GSK3 biomarkers in PBMCs, hippocampus and cortex, one (Arm 1) where we compared GSK3 biomarkers in PBMCs between volunteers with MCI and age-matched controls, and one (Arm 2) where we performed a step-wise increasing lithium dosing experiment in MCI volunteers over 12 weeks.

## Materials and Methods

### Materials

Vacutainers for blood collection were purchased from BD (Wokingham, United Kingdom) (367869), Optiprep media (D1556), lithium chloride (203637), and sodium chloride (S7653) were from Sigma (Gillingham, United Kingdom), Mono-Poly resolving media from MP Biomedicals (Santa Ana, United States) (0916980). Protease inhibitor tablets were from Roche (11836170001) and Bradford protein assay reagent was from Biorad (Watford, United Kingdom) (500-0006). The nitrocellulose (Amersham Protran) membrane and Phosphocellulose P81 paper were from GE Healthcare Life Sciences (Little Chalfort, United Kingdom) (10600002), the Enhanced Chemiluminescence (ECL) Kit used was from Amersham (RPN2209) and CL-Xposure film from Thermo Scientific (Waltham, United States). The radioisotope, γ-32P-ATP was purchased from Perkin Elmer (Waltham, United States). All other chemicals were of the highest grade available. All antibodies used in this study are detailed in Table 2.

**Table 2:** Antibodies used in this work.

Antibody	Manufacturer	Cat no	Dilution (1% BSA in TBST)	Secondary (in TBST)
Anti-Actin	Sigma	A3853	1:5000	Mouse
Anti-β-Catenin	Cell Signaling	2698	1:1000	Mouse
Anti-phospho-β-Catenin	Cell Signaling	9561	1:1000	Rabbit
Anti-c-Myc	Abcam	AB32	1:1000	Mouse
Anti-phospho-CRMP2 (Ser509/514)	In house		1:1000	Sheep
Anti-phospho-CRMP2 (Ser522)	In house		1:1000	Sheep
Anti-total CRMP2	Cell Signaling	9393	1:1000	Rabbit
Anti-phospho-CRMP4 (Ser509)	In house		1:1000	Sheep
Anti-total Glycogen synthase	Cell Signaling	3886	1:1000	Rabbit
Anti-phospho-Glycogen synthase	Cell Signaling	3891	1:1000	Rabbit
Anti-GSK3 α/β(for IB)	Cell Signaling	5676	1:1000	Rabbit
Anti-phospho GSK3 α/β (S21/9)	Cell Signaling	8566	1:1000	Rabbit
Anti-GSK3α (for IP)	Abcam	Ab40870	1 μg/0.1mg of lysate	Rabbit
Anti-GSK3β (for IP)	In House		1 μg/0.1mg of lysate	Sheep
Anti-total ERK (p44/42 MapK)	Cell Signaling	9102	1:1000	Rabbit
Anti-phospho-ERK (Thr202/Tyr204)	Cell Signaling	9101	1:1000	Rabbit
Anti-total PKB	Cell Signaling	2920	1:1000	Mouse
Anti-phospho-PKB (Thr308)	Cell Signaling	9275	1:1000	Rabbit
Anti-phospho-PKB (Ser473)	Cell Signaling	9271	1:1000	Rabbit
Anti-S6	Cell Signaling	2217	1:1000	Rabbit
Anti-phospho-S6 (Ser240/244)	Cell Signaling	2215	1:1000	Rabbit
Anti-Tau5	Millipore	MAB361	1:1000	Mouse
Anti-phospho-Tau (Thr231)	Millipore	AB9668	1:1000	Rabbit
Anti-phospho-Tau (Ser396)	Cell Signaling	9632	1:1000	Mouse
VASP	In house		1:1000	Sheep
HRP conjugated anti-RABBIT IgG	Thermo Fisher	S1440	1:2500	N/A
Anti-RABBIT IgG (H&L) IRDye800^®^Conjugated	Rockland	613-132-122	1:10000	N/A
Anti-RABBIT IgG (H&L) IRDye800^®^Conjugated	Licor	925-32211	1:5000	N/A
Anti-SHEEP IgG (H&L) IRDye800^®^Conjugated	Rockland	613-431-002	1:10000	N/A
Anti-MOUSE IgG (H+L) Alexa Fluor 680	Life Technologies	A21057	1:5000	N/A

### Animals

All experiments were performed using male Wistar rats (Harlan Ltd., United Kingdom), initial body weight 150–175 g. Animals were housed in cages of four (12:12 h light: dark cycle) at an ambient temperature of 22 ± 1°C and 50% humidity, with *ad libitum* access to food and water. All animal procedures were approved by the University of Dundee Ethical Review Process and were performed in accordance with UK Home Office regulations (under the auspices of Project License PIL60/4280). Animals were assigned at random to one of three treatment groups (i) Control (Saline), (ii) Low dose lithium chloride (2 nmoles/kg i.p.), or (iii) High dose lithium chloride (4 nmoles/kg i.p.). 3 h post injection animals were sacrificed by cervical dislocation and blood samples collected by cardiac puncture for the preparation of PBMCs and plasma lithium analysis. Brain regions (hippocampus, hypothalamus, cortex, and cerebellum) and liver samples were isolated and snap frozen in liquid nitrogen for subsequent biochemical analysis.

### Rodent Lithium Assessment

One milliliter of whole rat blood was collected from each animal on Sodium Heparin. Samples were spun and serum transferred to 1.5 ml Eppendorf tubes prior to running on an AVL 9180 Electrolyte Analyser, alongside an appropriate lithium standard curve.

### Rodent PBMC Collection

Peripheral Blood Mononuclear cells were collected from the rat blood samples immediately after collection using OptiPrep density gradient medium using the flotation technique as per manufacturer’s instructions. Protein was harvested from the cell pellet in lysis buffer [25 mM Tris–HCl (pH 7.4), 50 mM NaF, 0.1 M NaCl, 1 mM EDTA, 5 mM EGTA, 1% Triton-X, 10 mM Na-pyrophsphate, 0.1% beta-mercaptoethanol, 1 mM Na-vanadate, protease inhibitor cocktail tablet (Roche 11836170001) and 0.27M sucrose] and snap frozen in liquid N2 prior to storage at -80°C.

### Study Design

**FIGURE 1:**
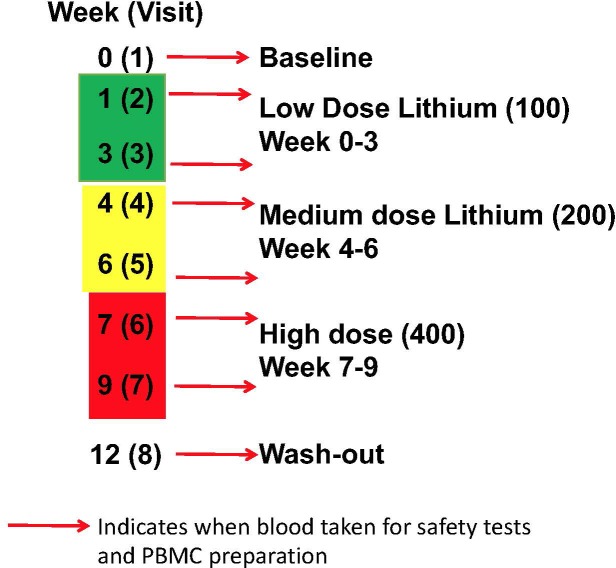
Lithium intervention Study Design (Arm 2). Eleven volunteers were given low dose (100 mg daily) for 3 weeks, then medium dose (200 mg daily) for 3 weeks, high dose (400 mg daily) for 3 weeks, prior to a 3 week washout period. Blood samples were taken for safety measures, PBMC preparation and Lithium measure at baseline (visit 1), 1 and 3 weeks after starting each dose (visit 2–7), and after washout (visit 8).

There were two arms to the human study, firstly a comparison of GSK3 biomarkers in PBMCs between volunteers with MCI and age-matched controls. This included a comparison of the biomarkers from the same individual in two samples taken 12 weeks apart. Secondly, an intervention arm, where volunteers with MCI were given lithium tablets for 9 weeks, with a step wise increase from 100 to 200 mg/d then 400 mg/d for 3 weeks each, followed by a 3 week wash out period (Figure 1). Blood was taken at baseline, 1 and 3 weeks after lithium dose change as detailed in Figure 1. The study was approved by the North of Scotland Research Ethics committee, number16/NS/006.

### Volunteer Recruitment and Definition of MCI

Participants over age 55 and with a diagnosis of MCI were recruited from Tayside Old Age Psychiatry and Geriatric Medicine outpatient clinics and by using Join Dementia Research (JDR) Database. Of 830 clinic contacts screened via consultant caseloads, 67 MCI patients were identified and invited by letter plus 4 contacts identified through existing local research lists and 3 via JDR. MCI was defined according to Petersen’s criteria (Petersen et al., 2001). Two brief standardized questionnaires [Bristol Activities of Daily Living Schedule and Geriatric Depression Scale Short Form (15 point version)] were administered to check diagnosis and existing cognitive assessments were documented from case file. The control group was recruited via two routes, firstly through Join Dementia Research and secondly by approaching spouses/partners/relatives of similar age to the MCI group but who lack an MCI diagnosis. Exclusions for the Study Arm 1 group were as follows; Dementia, Diabetes, current medication of Lithium or NSAIDS, BMI < 18 or > 30, incapacity to consent, Rheumatoid Arthritis and Autoimmune Inflammatory disorders and for Study Arm 2 the following additional exclusion criteria were applied: contraindication to Lithium Carbonate (Priadel) according to Summary of Product Characteristics, Moderate renal failure (defined by eGFR < 60 mL/min/1.73 m^2^) or severe Renal failure (defined by eGFR < 29 mL/min/1.73 m^2^), epilepsy, and suicidality. Screening included baseline blood tests for renal function and thyroid function.

Safety assessments were face to face and occurred at each scheduled visit at weeks 1, 3, 4, 6, 7, 9, and 12. Participants were asked about general health and the presence of any common side effects and a Lithium side effects rating scale (LISERS) completed where any side effects were reported. The blister pack was examined for compliance. Blood lithium concentration and renal function were assessed as standard clinical safety measures in line with guidelines for routine clinical practice. Lithium > 0.7 mmMol/L or significant deterioration in renal function (defined by eGFR falling to < 60 mL/min/1.73 m^2^) or acute kidney injury [diagnosed by acute rise in creatinine blood result according to NICE Guidelines for Acute Kidney Injury (Levin et al., 2008)] would result in withdrawal from lithium and entry to washout.

### Human PBMC Collection

Volunteer blood (13–18 ml) was collected in Na-heparin containing vacuettes, kept at room temperature and processed within 6 h of collection. In sterile test tubes 3.5 ml of blood was carefully layered on top of 3 ml Mono-poly resolving media. These were centrifuged for 45 min, 800 ×*g* in a swinging bucket rotor at room temperature without brake (Beckman Coulter Allegra X-12 centrifuge). The plasma layer at the top of the tube was then removed and discarded using a Pasteur’s pipette and the top layer of cells containing the mononuclear leucocytes was collected and combined in a clean test tube. The cell suspension was washed with three volumes of PBS and centrifuged 10 min, at room temperature, 250 ×*g* with brake on. The cell pellet was washed in PBS and centrifuged again. An equal volume of PBS was added to the pellet, the cells were resuspended gently and the cell mixture transferred to a cryovial and snap frozen in liquid N2 prior to storage at -80°C.

### Cell Lysis and Western Blot Analysis

The PBMC pellets were lysed on ice in lysis buffer. Following centrifugation to remove insoluble material, supernatants were collected and protein concentrations were determined using the Bradford method. Lysates were made up to equal concentrations with lithium dodecyl sulfate sample buffer added and subjected to SDS-polyacrylamide gel electrophoresis on 4–12% NuPAGE polyacrylamide gels (Novex, Life Technologies), then either stained using Coomassie Blue (SimplyBlue^TM^Safe Stain, Invitrogen, CA, United States), or transferred to nitrocellulose membrane. Membranes were blocked in 5% (w/v) BSA, incubated with primary antibodies overnight at 4°C, and then Alexa-fluor secondary antibodies for 1 h at room temperature. Proteins were visualized using the Licor Odyssey. When probing for phospho Akt(Ser473), HRP secondary antibody was used, detection achieved with ECL and proteins visualized on film using different exposure times, typically between 10 s and 10 min.

### Quantification of Immunoblots

Protein bands were quantified using the Image Studio software (Licor). Odyssey images were quantified directly while ECL images on film were scanned using a desktop scanner and imported into the software. The background was determined around each box individually, either left and right, top and bottom, or both with varying width, dependent upon the layout of each blot, but kept consistent per blot. Normalization was done by generating a ratio against a loading control (usually actin), or for Phosphorylation sites a ratio against the total protein signal of the protein of study where antibodies were available. The differences between groups were determined by using a Student *t* test. Analyses were performed using Excel (Microsoft Corp.).

### Sequential Immunoprecipitation and Assay of GSK3 Isoforms

A total of 200 μl of (50:50) Protein A agarose beads in PBS were conjugated to 20 μg of either anti-GSK3β or anti-GSK3α by incubating for 1 h at room temperature with shaking. Unbound antibody was removed by washing with lysis buffer and 5 μl of the (50:50) anti-GSK3β-beads incubated with 100 μl of 1 μg/μl cell lysate overnight at 4°C with shaking. GSK3β immunocomplexes were isolated by centrifugation at 3000 ×*g* for 5 min. Supernatant was incubated with 5 μl of (50:50) anti-GSK3α-beads for 4 h at 4°C prior to isolation of the GSK3α immunocomplexes by centrifugation at 3000 ×*g* for 5 min. All immunocomplexes were washed with 1 ml of lysis buffer, followed by 1 ml of kinase assay buffer (25 mM TRIS, 0.1 mM EDTA, 5% glycerol) containing 0.5 M NaCl and then 1 ml of kinase assay buffer before resuspending to 10 μl in assay buffer.

Each washed immunocomplex was incubated with PGS2 peptide (10 μM final), MgCl2 (10 mM final), γ - 32P-ATP (0.1 mM final, 0.5 × 10^6^ CPM/nmole) in assay buffer in a final volume of 50 μl at 30°C for 20 min. The assay was stopped by spotting the assay mix onto P81 phosphocellulose paper and placing in 10 mM Phosphoric Acid (PA). Papers were washed 4× 5 min in PA then once in acetone and air dried. Phosphate incorporation into the GSK3 substrate was quantified by counting the paper in a scintillation counter. One unit of activity of each protein kinase was calculated as the amount required to transfer 1 nmole of phosphate/min. The differences between groups were determined by using a Student *t* test. Analyses were performed using Excel (Microsoft Corp.).

## Results

### Clinical Studies

#### Recruitment of Volunteers With a Diagnosis of Mild Cognitive Impairment (for Study Arms 1 and 2)

**FIGURE 2:**
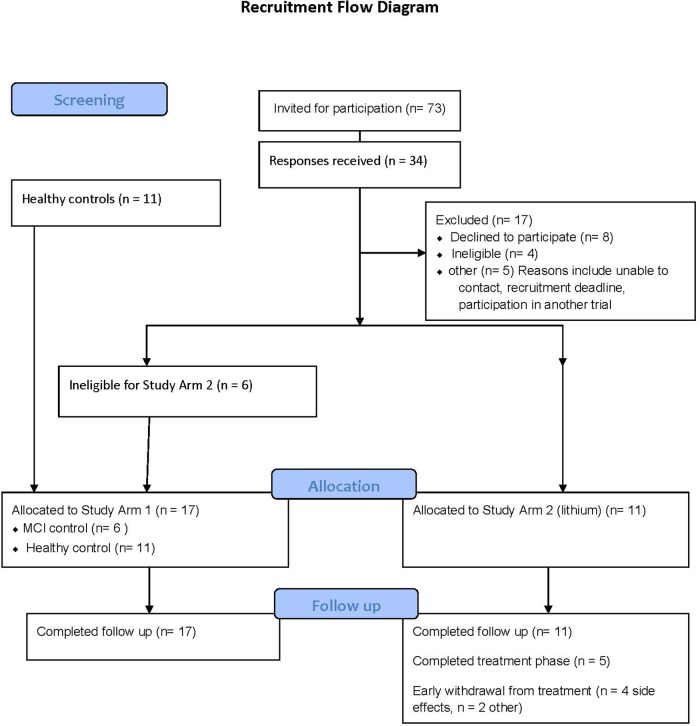
Consort diagram detailing the recruitment process for the clinical studies.

The recruitment strategy is provided in Figure 2. Study Arm 1 contained six MCI and eleven controls (all providing two blood samples, 12 weeks apart), and Study Arm 2 recruited eleven MCI volunteers for lithium treatment (eight blood samples across 12 weeks). The number of participants excluded due to BMI above 30 was greater than anticipated from this age group and was the most common reason for exclusion.

#### Retainment and Safety

There were no retainment issues in Study Arm 1, with no withdrawals and no adverse events reported. In contrast, of the eleven participants in Arm 2, six withdrew from the lithium titration phase (54%) due to reported side effects (*n* = 4), non-compliance (*n* = 1) and altered blood results (*n* = 1). All participants completed a lithium wash out visit (>3 weeks after last lithium dose). Interestingly, there was no association between lithium blood concentration and withdrawal (withdrawals had plasma lithium in the range 0.2–0.61 mmol/L, mean 0.38 mmol/L, while non-withdrawals plasma lithium ranged from 0.33–0.54 mmol/L, mean 0.42 mmol/L). The mean duration of lithium titration before withdrawal was 5 weeks (note; high dose lithium only started in week 6). Table 3 provides the plasma lithium of all participants through the study period, including those that withdrew. Five of the volunteers provided the full set of seven blood samples for lithium measure (Table 3).

**Table 3:** Plasma lithium level (mmoles/l) during stepwise increase in lithium dose in volunteers.

		Patient ID										
	Visit	20	29	19	24	15	14	16	10	30	32	21
Low dose	2 (week 1)	<0.2	<0.2	<0.2	?	**<0.2**	**<0.2**	**<0.2**	**<0.2**	**<0.2**	<0.2	<0.2
	3 (week 3)	<0.2	<0.2	<0.2	?	**<0.2**	**<0.2**	**<0.2**	**<0.2**	**<0.2**	<0.2	<0.2
Medium dose	4 (week 4)	<0.2	0.3	w	w	**<0.2**	**<0.2**	**0.24**	**<0.2**	**<0.2**	0.23	0.4
	5 (week 6)	0.23	0.33	w	w	**<0.2**	**<0.2**	**0.3**	**<0.2**	**<0.2**	0.45	<0.2
High dose	6 (week 7)	<0.2	0.61	w	w	**0.39**	**0.4**	**0.37**	**0.36**	**0.33**	w	<0.2
	7 (week 9)	w	0.44	w	w	**0.38**	**0.33**	**0.54**	**0.36**	**0.47**	w	w
	8 (week 12)	<0.2	NA	<0.2	<0.2	**<0.2**	**<0.2**	**<0.2**	**<0.2**	**<0.2**	<0.2	<0.2

*The 11 participants in Study Arm 2 had plasma lithium levels measured at baseline (visit 1) then after 1 and 3 weeks of each lithium dose, and once at least 3 weeks after stopping lithium (washout). Two volunteers withdrew (w) while on low dose (19 and 24), two on medium dose (21 and 32), and one did not take lithium as asked (volunteer 20). Low dose did not generate detectable lithium levels (0.2 assay detection limit), medium dose raised plasma lithium in four out of eight volunteers to detectable levels, with 0.45 mmoles/l the highest reported level at this dose (32). Finally, high dose raised plasma levels in every volunteer remaining in the study (29,15,14,16,10, and 30), with an average of 0.415 mmoles/l and a range of 0.33–0.61. All lithium levels returned to baseline within 3 weeks of discontinuation. Bold values indicate the completion of the study.*

There were no serious adverse events reported. Six participants experienced side effects (54%) with two of these continuing with the lithium titration phase to completion. Side effects reported at either moderate or severe intensity included: fatigue, metallic taste, dry mouth, nausea, decreased concentration, insomnia, or confusion. Feeling drowsy or lethargic during the day was the most commonly reported side effect with five of the six participants reporting this in either mild, moderate or severe intensity.

Only one individual of the eleven given lithium exhibited a change in renal function (mild reduction in eGFR to 57 mls/min with accompanying mild increase in creatinine). Examination of the case files revealed that while normal at trial initiation, this individual had previously had similar mild impairment of renal function on blood testing. There were no instances of electrolyte disturbance or other renal problems during the trial.

### Biomarker Studies

#### GSK3 Biomarkers in Rodent PBMC and Association With Neuronal GSK3 Activity

Our initial study was to investigate whether the GSK3 biomarkers in Table 1 could be detected in a PBMC preparation from rats, and whether detectable markers respond to lithium administration. Additionally, we wanted to try to investigate whether any lithium induced biomarker responses in the PBMCs associated with changes in the brain. Ultimately, we wanted to generate a focussed, validated, panel of GSK3 biomarkers as a readout for GSK3 function in humans to investigate early dementia, and for establishing efficacy of lithium dose.

**FIGURE 3:**
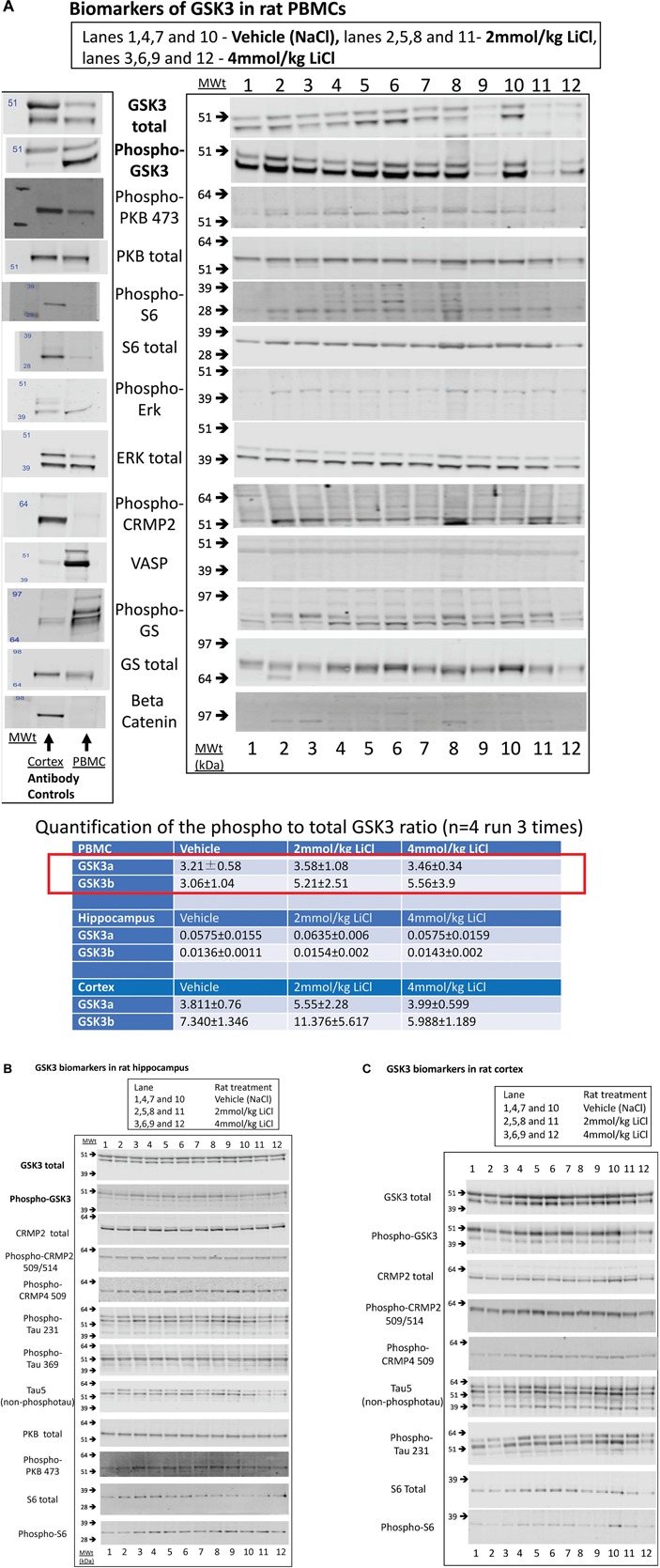
Biomarkers of GSK3 in rat tissues following lithium injection. Rats were given an i.p. injection of vehicle or lithium chloride as detailed in section “Materials and Methods,” and tissues isolated 3 h later. Representative examples of Western blots of rat protein lysates isolated from **(A)** rat PBMCs, **(B)** Hippocampus, or **(C)** Cortex, are provided using the antibodies stated. In **(A)**, we also provide a comparison of vehicle treated rat PBMC lysate with rat brain cortex lysate with every antibody as evidence for the utility of each antibody (antibody control). The ratio of expression of GSK3 phosphorylation at the N-terminal inhibitory site to the overall isoform expression was quantified from the images as detailed in section “Materials and Methods,” and is provided for each tissue (±SD) in the table inserted into **A**. The red box highlights the major biomarker changes in response to lithium, although only the P-GSK3β-total GSK3β ratio was significantly increased by lithium (*p* < 0.05, student *t* test).

However, we could detect only eight of the biomarkers from Table 1 in rat PBMCs either using immunoblotting, loading up to 50 μg of total protein on the gels, or by immunoprecipitation of GSK3 isoforms from cell lysates and assay (Figure 3A, for examples of immunoblots, and Table 4 for IP assay data). Importantly, only four of these biomarkers were detected robustly (signal > twice background), and reproducibly from multiple separate PBMC preparations (Table 5). These were Phospho-Ser21/9 of GSK3, total GSK3 (both by immunoblot, Figure 3A), and GSK3α and GSK3β activity (measured by IP-assay, Table 4). We are confident that the blotting reagents were suitable for detection of each protein (positive controls included in Figure 3A and biomarkers were detectable in brain tissues, Figure 3). Therefore, the major GSK3 regulatory pathways and the best characterized GSK3 substrates to date all proved difficult to detect robustly in PBMCs by immunoblotting.

**Table 4:** GSK3 specific activity (measured by IP assay) in rat tissue lysates.

	Vehicle	2 mmoles LiCl	4 mmoles LiCl	All LiCl samples
**PBMC**
GSK3a	2736 ± 752	3250 ± 805	3660 ± 736	3455 ± 798
GSK3b	9703 ± 640	8130 ± 611^∗^	8997 ± 895	8563 ± 880^∗^
**Hippocampus**
GSK3a	19497 ± 2077	17953 ± 984	19681 ± 2140	18817 ± 1877
GSK3b	15029 ± 1296	15135 ± 2689	14729 ± 1598	14932 ± 2221
**Cortex**
GSK3a	21260 ± 3052	20553 ± 5438	17391 ± 4674	18972 ± 5311
GSK3b	14554 ± 940	16175 ± 1262	14577 ± 2593	15376 ± 2190

*CPM/100 μg of tissue protein lysate ± SD, n = 4, ^∗^p < 0.05 vs. vehicle.*

**Table 5:** Expression of potential biomarkers of GSK3 function in rat tissues.

Biomarker	Expression in PBMCs	Expression in Hippocampus	Expression in Cortex	Response to lithium injection
Total GSK3α and β protein	+ + +	+ + +	+ + +	No
P-SER9/21 GSK3	+ + +	+ + +	+ + +	Yes, but not dose dependent and in PBMCs only.
P-SER389 GSK3	No	No	No	---------
P-TYR GSK3	+	+ + +	+ + +	No
GSK3α activity	++	+ + ++	+ + +	No
GSK3β activity	+ + +	+ + +	+ + +	Yes, but not dose dependent and in PBMCs only.
B-catenin	-	+ + +	-	
P-509CRMP2	-	++	++	No
P-509CRMP4	-	+ + +	+ + +	No
P-B-CATENIN	-	+	-	No
VASP	++	+	+	No
P-glycogen synthase	++	-	-	No
PTAU231	-	+ + +	++	No
PTAU396	-	++	+	No
TAU5	-	++		
P-ERK	+	+ + +	+ + +	
P-PKB473	+	+ + +	+ + +	No
P-PKB308	-	-	-	---------
Ribosomal S6	-	++	+ + +	No
Phospho-S6	+	++	+ + +	No

*+, ++, +++ represents increasing levels of detection, – represents very weak and inconsistent detection.*

We next investigated whether any of these markers were influenced by a single i.p. injection of LiCl vs. NaCl (control). 3 h after injection the blood lithium levels in the rats were raised in a dose dependent fashion, with roughly twice the plasma lithium concentration achieved by doubling the lithium dose (Table 6). Cell based studies had previously suggested that 0.5–1 mM Lithium is required to acutely reduce cellular GSK3 activity by around 50%, and we were achieving these levels in the rat blood with a single injection. Therefore, we monitored the potential GSK3 biomarkers in protein lysates isolated from PBMCs (Figure 3A) and two brain regions (Figure 3B,C), in order to assess the response to Lithium injection (summary in Table 5). In addition, we immunoprecipitated, and assayed the specific activity of the two GSK3 isoforms from the same tissues (Table 4), as lithium is reported to enhance covalent inhibitory modification of GSK3. The phosphorylation of Ser9 of GSK3β was enhanced in PBMCs by lithium injection (see insert table in Figure 3A for quantification of immunoblots), and consistent with this, the activity of GSK3β (but not GSK3α) was significantly reduced in PBMCs (Tables 4, 5). This indicates that a plasma lithium level of 0.83 mM was sufficient to enhance Ser9 phosphorylation and at least partly inhibit GSK3β activity, and could be achieved by this single lithium injection. Interestingly, double the plasma level of lithium (1.6 mM) did not further increase the degree of Ser9 phosphorylation nor enhance the inhibition of GSK3β, suggesting the effect had plateaued. In addition, this plasma level of lithium did not significantly alter any of the measurable GSK3 biomarkers studied in the hippocampus, or cortex (Figure 3B,C, quantified in table within Figure 3A). This may mean the brain does not get exposed to the same level of lithium as the blood, or the time point we examined was not optimal for effects on the brain.

**Table 6:** Rat plasma lithium levels achieved after a single i.p. dose of lithium chloride.

Rat number	Lithium dose	Plasma lithium (mmoles/l)	Averages
1	Vehicle(NaCl)	Not detectable (0.15 detection limit)	
4	Vehicle	Not detectable	Not detectable
7	Vehicle	Not detectable	
10	Vehicle	0.17	
5	2 mmoles/kg	0.39	
2	‘’	0.83	
11	‘’	0.87	0.83 mM
8	‘’	1.23	
6	4 mmoles/kg	0.94	
9	‘’	1.02	
3	‘’	1.78	1.635 mM
12	‘’	2.8	

#### GSK3 Biomarkers in Human PBMCs

The next step in our study examined the GSK3 biomarkers, identified in the rat study, in PBMCs isolated from human volunteers. In Study Arm 1 we wanted: (1) to establish that the GSK3 activity biomarkers detectable in rat PBMCs could also be detected in cells isolated from the human population of greatest interest (i.e., MCI), (2) to investigate the reproducibility of marker detection in two PBMC preparations generated from the same individual but taken 12 weeks apart, and finally, (3) to ask whether there were major differences in any of these markers that associated with MCI diagnosis. We recruited six patients with MCI (mean age 78, range 77–81) and eleven non-MCI, age-matched controls (mean age 76, range 65–84) to this arm. In addition, we had baseline samples from the 11 MCI volunteers recruited to Arm 2 below (making *n* = 17 for the MCI group).

**FIGURE 4:**
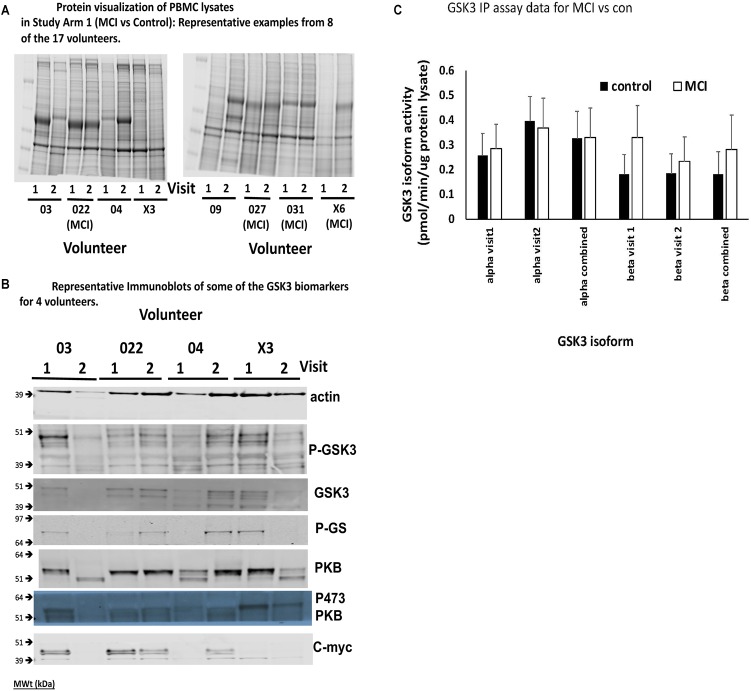
Comparison of Biomarkers of GSK3 in human PBMCs isolated from volunteers with MCI and controls (Study Arm 1). Protein lysates were prepared from PBMCs isolated from two groups of human volunteers, one with a diagnosis of MCI, the other age matched but with no MCI. In each case the volunteer provided two blood samples for PBMC preparation, taken 12 weeks apart (Visit 1 and 2). **(A)** Representative Coomassie protein staining of PBMC lysates from 8 of the 17 volunteers in Study Arm 1, where equal total protein was loaded on the gel. **(B)** Representative immunoblots of biomarkers within the PBMCs of four of the volunteers in Study arm 1. **(C)** Immunoprecipitation and assay of GSK3 isoforms from 50 μg of protein lysate in each of the PBMC samples. The specific activity was quantified for GSK3alpha and GSK3beta from all PBMC preparations and the average activity (±SE) for the MCI and control groups at each visit, as well as the two visits combined, is given.

A major finding of this work was that the consistency of protein recovery and quality between the human PBMC preparations, even those generated from samples obtained from the same individual, was varied (Figure 4A for protein stains). In some cases, total protein recovery was relatively low (e.g., visit 1 volunteer X6), in others there was clear evidence of substantial degradation (e.g., visit 2 volunteer 03, visit 1 volunteer 04, visits 1 and 2 volunteer X3, visit 2 volunteer 09). The Coomassie stained gels emphasized that 13 of the 45 PBMC isolates investigated had noticeable overall protein degradation. Nevertheless, we went on to examine several of the biomarkers from Table 1, in particular the four which were robustly detected in rat PBMCs. There was also variability in the biomarkers across the samples, even between samples from the same individuals, mostly associated with protein quality (Figure 4B), however, we were able to confirm which biomarkers could be detected in human PBMCs. In addition to phosphorylation of Ser9/21 of GSK3, total GSK3 and the activity of the GSK3 isoforms, we could also detect the GSK3 substrate c-myc (Figure 4B). Due to the poor quality of protein isolation we quantified the protein staining in every sample (22 healthy and 12 MCI in Study arm 1, plus the baseline PBMC preparations from the 11 MCI volunteers in Study Arm 2 below, so *n* = 22 and 23, respectively), and discarded any samples with less than 20% of the average protein level by protein staining (Figure 4A, e.g., visit 1 volunteer X6), or samples with < 10% of the average actin staining (Figure 4B, e.g., visit 2 Volunteer 03). We then quantified the immunoblot densities for the remaining samples (control group *n* = 14, MCI group *n* = 18), for every detectable biomarker. The phosphorylation of GSK3β at Ser-9 (47 kDa) and the activity of GSK3b were the only biomarkers that were significantly affected by lithium in the rat study (Figure 3A). In the human PBMCs we generated a P-GSK3β to actin ratio for every sample. The healthy control samples had a P-GSK3β:actin ratio of 0.0283 ± 0.018 (*n* = 14) and the MCI samples had a ratio of 0.0295 ratio ± 0.015 (*n* = 18). These data sets were not significantly different (*p* > 0.05), implying that development of MCI does not alter this aspect of GSK3 regulation, at least in PBMCs. Similarly, an IP assay of GSK3 activity indicated a possible trend of increased GSK3β activity in the MCI group, however, this did not reach significance (Figure 4C). The GSK3 substrate, c-myc, was detected in human PBMCs (Figure 4B), however, there was not a significant difference in the c-myc:actin ratio between controls and MCI (0.143 ± 0.1 controls, vs. 0.129 ± 0.1 MCI, *p* = 0.77). This protein is degraded upon phosphorylation by GSK3, hence the data would suggest no significant increase in GSK3 activity in MCI PBMCs.

#### Does Lithium Affect GSK3 Biomarkers in Human PBMCs

**FIGURE 5:**
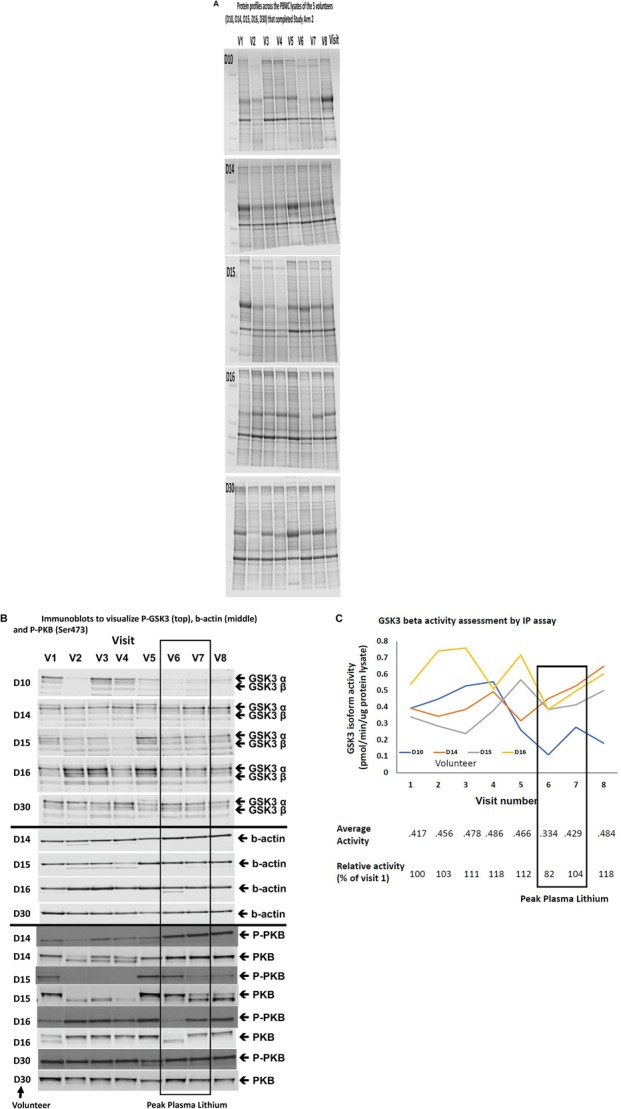
Comparison of Biomarkers of GSK3 in human PBMCs isolated from volunteers with MCI treated with a stepwise increasing dose of Lithium (Study Arm 2). Protein lysates were prepared from PBMCs isolated from volunteers with MCI who underwent lithium treatment (see Figure 1). **(A)** Protein profiles (Coomassie staining) from the PBMC lysates of the five volunteers that completed eight visits. Equal amounts of protein were loaded in each lane (by Bradford assay). **(B)** Immunoblot of all of the lysates from the same five volunteers using the phosphoser9/21 GSK3 antibody. The box around visit 6 and 7 highlights the samples with the peak plasma lithium levels (Table 3). **(C)** GSK3β isoform specific activity in PBMCs isolated across the eight visits for four of the five volunteers that completed the study. The average GSK3β activity for each visit and the average GSK3β activity relative to the baseline activity for each volunteer are given under the graph. The box highlights samples (6 and 7) with the peak plasma lithium levels (Table 3).

Although the GSK3 biomarkers were not significantly altered in PBMCs in people with MCI we proposed that they could still be of use to monitor GSK3 activity status in response to interventions which work through modulating GSK3, such as lithium. Therefore, we performed study Arm 2 generating up to eight different PBMC preparations from each of the 11 volunteers, taken at various stages during a step wise increasing lithium dosing schedule (Figure 1). Separate blood samples were taken at every time point to measure plasma lithium and isolate PBMC preparations, respectively, and so biomarker detection in the PBMC preparations could be linked to changes in blood lithium (Table 3). Two volunteers withdrew while on low dose (ID 19 and 24), two on medium dose (ID 21 and 32), one did not take the lithium (ID 20) and one failed to attend on more than one occasion. This left five volunteers who completed the full 8 sample set (Figure 1, 2). Low dose lithium did not generate detectable plasma lithium levels (Table 3), medium dose raised plasma lithium to detectable levels in only four out of the eight volunteers that remained at this stage, with 0.45 mmol/l the highest reported level on medium dose (ID 32). Finally, high dose treatment raised plasma levels in every volunteer (ID 29,15,14,16,10, and 30), with an average of 0.415 mmol/l and a range of 0.33–0.61 in these six volunteers (Table 3). All lithium levels returned to baseline within 3 weeks of discontinuation. Five volunteers (ID 10, 14, 15, 16, and 30) provided all eight PBMC preparations (baseline, six samples during dosing and a final washout sample, Figure 1). The PBMC preparations had significant variation in the quality of protein recovery, even across the samples isolated from the same individual (Figure 5A), however, this was not related to the lithium dose at time of blood collection. We examined the expression of the GSK3 biomarkers detectable in rat and human PBMCs, across these eight samples for each of the five volunteers. Quantitation of these immunoblots was problematic due to the high variability in loading controls (despite matching loading for total protein), which implies protein degradation or dephosphorylation in many of the samples. Indeed, it is worth noting that the variability and quality of the P-GSK3 and P-PKB signals in Figure 5B were very sensitive to even minor variations in protein (Figure 5A) and housekeeping protein (Figure 5B, actin). For example compare protein, b-actin, P-GSK3 and P-PKB in volunteer D15, visit 2, 3, and 4 (protein and b-actin degradation) with volunteer D14, visit 6, 7, and 8. Therefore, although we found no significant effect of lithium on the phosphorylation of GSK3 and PKB (Figure 5B and quantified in Supplementary Table), or the activity of either GSK3 isoform (GSK3 beta activity shown in Figure 5C, *p* > 0.05), the extensive variation in human PBMCs may have greatly reduced the power to detect effect sizes of those seen in the rat study. It should be noted that the highest blood lithium levels (visits 6 and 7 in Figure 5) never reached the lithium levels in the acute dosing rat study, and this implies that 0.33–0.61 mM plasma lithium is not sufficient to regulate the inhibitory phosphorylation of GSK3β in human PBMCs.

These data emphasize the need to improve the standard PBMC isolation process, identify further GSK3 activity biomarkers, and increase volunteer numbers to utilize PBMC preparations for monitoring GSK3 activity.

## Discussion

This was a preliminary study to investigate the feasibility and methodology around performing GSK3 functional biomarker development in a highly relevant human population. The study had several research goals, firstly to validate which of the numerous potential biomarkers of GSK3 biology could be detected in PBMCs, secondly to establish whether expression of these detectable biomarkers was affected by a diagnosis of MCI, thirdly, to investigate whether expression of these biomarkers was modified by treatment with lithium, at a dose up to that used to treat Bipolar disorder, and finally to add to the growing information on tolerance of lithium in the MCI population. The hope was that we would generate information to inform the design of a larger clinical assessment of lithium treatment for the prevention of progression from MCI to dementia (Sutherland and Duthie, 2015). Validated biomarkers of GSK3 function in human PBMCs could have potential use in multiple other conditions, including diabetes, inflammation, and cancer (Wagman et al., 2004; Hernandez et al., 2009; Castillo-Quan et al., 2016; Jope et al., 2017; Robertson et al., 2018). GSK3 inhibition is considered to have potential therapeutic value in all of these conditions, therefore robust, easily attainable markers of GSK3 may have clinical utility in diagnosis, prognosis and response to therapy (such as response to lithium).

Although there are a host of potential biomarkers for GSK3 function in cells and tissues, none are, in themselves, ideal for accurate and complete assessment of GSK3 activity. The main biomarker used in numerous studies is the phosphorylation status of Ser21 of GSK3α, and the equivalent residue Ser9 in GSK3β (Hye et al., 2005; Li et al., 2007; Soutar et al., 2010). However, while this modification is associated with GSK3 activity (Sutherland et al., 1993) it is a semi-quantitative, comparative measure and only assesses GSK3 activity toward primed substrates (Frame et al., 2001), which may not represent all of the clinically relevant GSK3 substrates (e.g., tau). Importantly, it does not reflect GSK3 regulation by other mechanisms, including by small molecule inhibition. Although some small molecule inhibitors such as lithium do enhance this phosphorylation it is unlikely to be as potent an inhibitory mechanism as direct inhibition through the active site of GSK3, and the two mechanisms may function at distinct lithium concentrations. Therefore, monitoring this phosphorylation site provides incomplete information on overall GSK3 regulation and function. Additional approaches include measuring upstream regulatory pathways, such as the PI3K-Akt pathway which regulates cellular GSK3 in response to insulin and some growth factors. However, other pathways including the Ras-MAPK, PKA and mTOR-S6K can complicate the input of the PI3K-PKB regulation (Eldar-Finkelman et al., 1995), and the degree of upstream pathway activation does not necessarily reflect the degree of GSK3 inhibition. Therefore, we believe that the most accurate approach is to combine assessment of the Ser21/9 phosphorylation status, the inherent activity of GSK3 isoforms (by IP assay), while simultaneously measuring the phosphorylation of a range of substrates known to be directly targeted by GSK3. This would provide a more quantitative and complete assessment of GSK3 functional status. Indeed, many of the targets of GSK3 become targets for the β-TrCP ubiquitination system, thereby directing them to the proteasome for destruction (Robertson et al., 2018). Therefore, there may be a panel of GSK3 targets whose protein levels provide a functional measure of GSK3 activity steady state level. What is clear is that in order to develop accurate tools to monitor GSK3 activity in clinical trials we need validated, selective and sensitive biomarkers of GSK3, either in tissues of importance to disease or in surrogate tissues (such as blood cells). This was the starting point for our investigation of the range of potential biomarkers of GSK3 function listed in Table 1.

### Limitations of the Current Study

Unfortunately, our study failed to achieve the desired outcome of establishing a panel of biomarkers for GSK3 that could be used to track early disease or drug response, and determine the appropriate dose of lithium for inhibiting GSK3 in MCI volunteers. This was mainly due to the unexpected poor quality of biomarker detection from PBMCs, which meant our studies were underpowered. However, we established a number of methodological issues and the data does question the clinical utility of most of the biomarkers listed in Table 1. Firstly, very few of them were detectable (by immunoblot) in PBMCs in rats or humans. Secondly, and probably most importantly, the quality and reproducibility of the signaling proteins (especially phosphoproteins) isolated from human PBMCs using standard protocols was disappointingly poor. Finally, our study in the rats indicated that there may be a complex non-linear relationship between plasma lithium level, GSK3 regulation in PBMCs and GSK3 regulation in brain.

Most of the biomarkers from Table 1 are only readily detectable in tissues where their function is important, (e.g., glycogen synthase is most highly expressed in muscle or liver tissues, while tau is neuronally enriched), and as such it is perhaps not surprising that many are not highly expressed in blood cells. The list of potential “GSK3 biomarkers” is actually much greater than that listed in Table 1, with more than 100 substrates of GSK3 reported in the literature (Sutherland, 2011). However, those in Table 1 are the best validated targets, and/or those with the most sensitive and specific antibodies, to facilitate detection by immunoblotting. We propose that it is now time to widen the search for well validated GSK3 targets to aid the development of a “GSK3 biomarker panel” for application to multiple tissues and species (Robertson et al., 2018), and it may be sensible to move to quantitative proteomic technology rather than relying on immunoblotting.

We would also propose that improved protocols to isolate human PBMCs (or other blood cells) are required to permit quantification of metabolic signaling pathways. Molecules such as GSK3 are regulated by a wide variety of extracellular cues. This may include some of the chemicals involved in PBMC isolation, or volunteer specific characteristics such as hormonal or diurnal cycles and dietary or stress influences on the day of blood collection. Indeed, the high degree of variation in our human PBMC preparations compared to the rat preparations would point to these latter influences as worthy of consideration.

Previously, increased GSK3 protein levels were found in white blood cells in AD and MCI patients (Hye et al., 2005), while reduced GSK3 phosphorylation was found in platelets of MCI patients (Forlenza et al., 2011b). We were not able to confirm these important findings possibly due to the poor quality of our PBMC protein preparations. It is also worth noting that the relationship between plasma lithium and GSK3β phosphorylation and activity in rat PBMCs was unusual. There was a similar effect on GSK3 inhibition at 0.83 and 1.6 mM plasma lithium. This may mean that the maximal dose dependent effect on GSK3β phosphorylation was below 0.83 mM, however, the degree of effect on GSK3 was not major (<twofold enhancement of phosphorylation and around 15% inhibition). This may be related to the fact we examined a single time point or the nature of this biomarker (phosphorylation rather than direct measure of GSK3 activity). For example, if GSK3β was 50% phosphorylated in control animals then the maximum possible effect of lithium on phosphorylation would be a twofold increase. As stated earlier, the IP assay is perhaps not the ideal biomarker for effects of small molecules, as lithium would be washed away during the IP process but it should still assess activity changes due to the inhibitory phosphorylation. This is why we believe that the accurate assessment of the pharmacological relationship between plasma lithium and GSK3 regulation would be best achieved with biomarkers that directly monitor GSK3 specific targets rather than simply the phosphorylation of GSK3.

It was concerning that we did not detect any effects of lithium on any GSK3 biomarkers in the brain of the rats despite achieving relatively high plasma levels of 1.6 mM lithium (albeit acutely). In this case we were monitoring several markers that we know are modified by GSK3 inhibition in cell culture (Cole and Sutherland, 2008), and in animal models of GSK3 deficiency (Soutar et al., 2010). This implies that this acute level of plasma lithium is not sufficient to regulate GSK3 in these brain areas. Alternatively, it is possible that either the injection resulted in a very transient increase in plasma lithium that is not sufficient to alter these biomarkers, or that the time point after injection was not optimal for effects on brain biomarkers. In essence a much more extensive study is needed to truly establish the lithium dose required to modify GSK3 activity in these brain areas.

Despite these major technical issues around the assessment of current GSK3 biomarkers in human PBMCs we were encouraged by the interest of the MCI patient population, as well as the relative safety of lithium in this population. This is consistent with previous studies in MCI (Forlenza et al., 2011a,b) and other conditions (Li et al., 2007; Hampel et al., 2009; Diniz et al., 2011; McKnight et al., 2012), where lithium has been found to be more safe and certainly manageable than current perception may have us believe (Macdonald et al., 2008). We should also keep in mind that lithium is a low-cost drug and even continuous monitoring for the known side effects is relatively cheap. Therefore, we provide further evidence that preforming an efficacy trial on the effect of lithium on progression from MCI to dementia is not only feasible but worthwhile. Within that study one could expand the investigation of GSK3 biomarkers from a simple readout for diagnosis, or drug action, to investigate any association with conversion from MCI to dementia. As such we are actively working to address the technical issues listed above.

## Ethics Statement

The study was approved by the North of Scotland Research Ethics Committee, number 16/NS/006. All animal procedures were approved by the University of Dundee Ethical Review Process and were performed in accordance with UK Home Office regulations (under the auspices of Project License PIL60/4280).

## Author Contributions

CS, AD, JG, and SL raised the funding for the study. CS was the Chief Investigator, contributing to data collection, analysis, and writing of the manuscript. AD was the Principle Clinical Investigator in Dundee and contributed to writing of the manuscript. SL was the Principle Clinical Investigator in Oxford. AD, CM, SL, and JG were responsible for volunteer recruitment, clinical ethics, and volunteer safety monitoring as well as contributing to study design, clinical data analysis, and writing of the manuscript. LvA, AM, and JGall were responsible for all rodent and lab work, data storage, and contributed to the writing of the manuscript.

## Conflict of Interest Statement

The authors declare that the research was conducted in the absence of any commercial or financial relationships that could be construed as a potential conflict of interest.
