# Supplementary material for: Recruitment, Retainment, and Biomarkers of Response; A Pilot Trial of Lithium in Humans With Mild Cognitive Impairment
Source: Front Mol Neurosci. 2019 Jun 28;12:163. doi: 10.3389/fnmol.2019.00163 (PMC6610581; doi:10.3389/fnmol.2019.00163)
Supplement: Supplementary file 1 [file Data_Sheet_1.docx]

Supplementary Table- Quantification of the GSK3 and PKB immunostaining in Figure 5B (n=4 for each visit)

| **Visit Number** | **Lithium Dose** | **GSK3α:actin** | **GSK3β:actin** | **P-PKB-total PKB** |
| --- | --- | --- | --- | --- |
| 1 | Baseline | 0.75 | 0.11 | 0.74 |
| 2 | Low (week 1) | 0.77 | 0.34 | 0.37 |
| 3 | Low (week 3) | 0.78 | 0.39 | 0.38 |
| 4 | Medium (week 1) | 0.53 | 0.22 | 1.04 |
| 5 | Medium (week 3) | 0.59 | 0.19 | 0.92 |
| 6 | High (week 1) | 0.61 | 0.40 | 0.59 |
| 7 | High (week 3) | 0.63 | 0.38 | 0.90 |
| 8 | Washout | 0.61 | 0.35 | 0.60 |
